# Exploring park visitation trends during the Covid-19 pandemic in Hungary by using mobile device location data

**DOI:** 10.1038/s41598-023-38287-3

**Published:** 2023-07-08

**Authors:** György Csomós, Endre Márk Borza, Jenő Zsolt Farkas

**Affiliations:** 1 Department of Civil Engineering, Faculty of Engineering, University of Debrecen, 2-4 Ótemető út, Debrecen, 4028 Hungary; 2 CIAS, Corvinus University of Budapest, 8 Fővám tér, Budapest, 1093 Hungary; 3 Centre for Economic and Regional Studies, Great Plain Research Department, 3 Rakóczi út, Kecskemét, 6000 Hungary

**Keywords:** Socioeconomic scenarios, Environmental impact

## Abstract

Sweeping changes in park visitation have accompanied the Covid-19 pandemic. In countries where governments imposed strict lockdowns during the first wave, park visitation declined in cities. The benefits of visiting urban green spaces on people’s mental and physical health and well-being are generally acknowledged; many people in confinement during lockdowns reported increasing mental health issues. Therefore, based on lessons learned from the Covid-19 pandemic’s first wave, urban parks and other urban green spaces remained open in most countries in subsequent pandemic phases. Furthermore, many studies have reported an overall increase in park visitation after strict lockdowns imposed in the pandemic’s first wave have been removed. This study aims to investigate park visitation trends in Hungary based on a dataset of 28 million location data points from approximately 666,000 distinct mobile devices collected in 1884 urban parks and other urban green spaces in 191 settlements between June 1, 2019, and May 31, 2021. Findings demonstrate that park visitation increased in the inter-wave period of 2020, compared to the pre-pandemic period of 2019, and decreased in Waves 2–3 of 2021, compared to Wave 1 of 2020.

## Introduction

As 2020 began, the world faced a major global health crisis: the Covid-19 pandemic. It has caused the loss of almost 7 million lives as of February 2023. In response to Covid-19’s spread, countries have introduced various measures, including nationwide lockdowns, curfews, and travel restrictions. In addition, because the principal transmission method for Covid-19 is airborne respiratory particles or fluids^1^, many national and municipal governments shut down urban parks and other urban green spaces due to fears that they could facilitate the transmission of the virus^2–7^. As a result, during the first few months (i.e., the first wave) of the pandemic, cities worldwide experienced a significant decline in park visitation. Still, shutting down urban parks yielded controversial findings regarding their benefits, drawbacks, and other effects^8^. For example, many studies demonstrate the positive impact of urban green spaces on visitors’ physical and mental health and well-being^9–13^. Other studies, however, report that lockdowns may trigger mental health issues (e.g., many people describe increased depression and anxiety)^14,15^.

Accordingly, based on lessons learned from the first wave of the Covid-19 pandemic, urban green spaces remained open in most countries in subsequent pandemic phases. Many studies demonstrated an overall increase in park visitation during the Covid-19 pandemic (except for the first some months when total lockdowns were imposed) compared to the pre-pandemic year^16–20^. However, it would be a mistake to consider the post-first-wave period of the Covid-19 pandemic as homogeneous: Sequences of subsequent waves interrupted by inter-wave periods characterize it. These new phases have differed in detected cases and daily deaths and might have provoked different responses from national and municipal governments, significantly impacting park visitation trends. In addition, mass vaccination programs launched globally in 2021 have helped countries gradually remove restrictions.

Therefore, this paper will explore park visitation trends in Hungary during different phases of the Covid-19 and pre-pandemic periods. More specifically, we compare park visitation trends in the inter-wave period of 2020 with the same period of 2019 and the second and third waves of 2021 with the first wave of 2020. For the analysis, we use location data of 666,156 distinct mobile devices that produced approximately 28 million location data points in 2019–2021. Mobile device (e.g., smartphone) location data is often used to quantify and analyze human activity in urban spaces^21–25^. In addition, our dataset contains 1884 parks in 191 settlements in Hungary, allowing us to demonstrate park visitation trends in different pandemic phases by park category and settlement type.

Overall, we set the following research questions:

- How did park visitation trends change in different phases of the Covid-19 pandemic?
- Which park types witnessed the most dramatic changes in the number of visitors in different phases of the Covid-19 pandemic?
- How did park visitation trends change by settlement type in different phases of the Covid-19 pandemic?

This paper is structured as follows: after the introduction, we will present the materials and methods we used for the analysis. These sections will be followed by the Results section. Then we will discuss changes in park visitation by park category and settlement types in different pandemic phases. We will also note the limitations of the research. Finally, we will draw the conclusions.

## Materials and methods

In this paper, we investigate park visitation trends in four different periods of the Covid-19 pandemic in Hungary. To do this, we use mobile device location data covering two years (June 1, 2019–May 31, 2021). Therefore, in the Materials and methods section, we will discuss the research’s three main dimensions: (1) the Covid-19 pandemic in Hungary, (2) the classification of urban parks, and 3) the mobile device GNSS-location database.

### A brief chronological overview of the Covid-19 pandemic in Hungary

When Italy experienced a significant outbreak at the beginning of 2020^26^, the Hungarian government gradually introduced measures to prevent Covid-19 from entering its borders for as long as possible. However, despite all efforts against its infiltration, the first Covid-19 case in Hungary was detected on March 4. A week later, the government officially declared a state of emergency (see Supplementary Table S1). Thus, on March 27, with a nationwide curfew imposed, the first lockdowns began. In Hungary’s first wave of the Covid-19 pandemic (March 4–May 24, 2020), local governments closed most of the urban parks. Only dog walking and individual sports (e.g., running and Nordic walking) were allowed in those that remained open. In the first wave, government officials and health professionals implored people to avoid personal contact, while the media launched a massive “Stay at home!” campaign. Parallel with the decline in daily deaths, the government gradually lifted curfews and finally removed all the restrictions on May 25.

In the summer of 2020, the number of detected cases and daily deaths had remained low; however, at the beginning of October, it became apparent that a second wave of Covid-19 would evolve. A dramatic surge in daily deaths was experienced at the beginning of November, forcing the government to introduce nationwide restrictions again. During the first wave (i.e., the second quarter), total lockdowns were imposed, resulting in a GDP fall of approximately 14%. So, to avoid pushing the economy into a recession, the government introduced restrictions gently.

**Figure 1:**
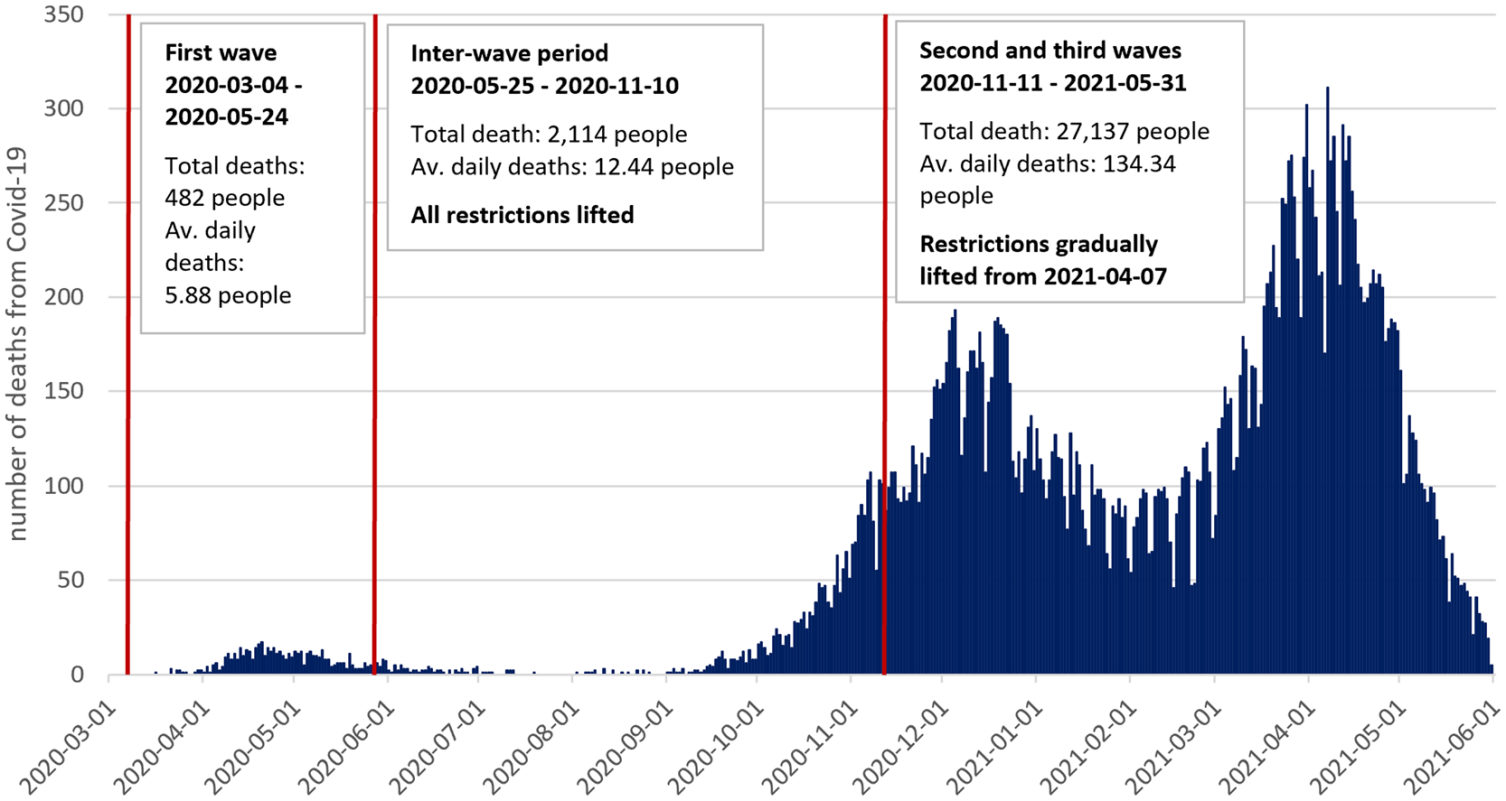
Covid-19 waves and death tolls in Hungary between March 1, 2020, and June 1, 2021. The first two years of the Covid-19 pandemic in Hungary can be divided into three phases. During Wave 1, the Hungarian government implemented total lockdowns. The inter-wave period spans the summer and most of the fall of 2020. During this phase, all restrictions were lifted. From the middle of October, a surge in deaths occurred, forcing the government to introduce partial lockdowns. Daily deaths decreased from mid-January 2021, eliciting hopes that the second wave of Covid-19 would ebb. However, before its end, deaths surged dramatically, reaching a peak of 311 deaths on April 7. As a result, the government implemented total lockdowns in Wave 3.

From November 11, a countrywide night curfew (between 8 p.m. and 5 a.m.) was in effect, and indoor facilities were ordered to close. In February 2021, parallel with the launch of a nationwide vaccination a decline in daily deaths occurred. However, threatening the government’s hopes of keeping the economy afloat, in March 2021, a dramatic surge in detected cases and daily deaths became apparent (marking the start of the third wave). As a result, on March 8, the second countrywide lockdown was enacted, closing kindergartens and elementary schools. In contrast to the first wave, urban parks remained open during this lockdown. In addition, health professionals urged people to go to parks, warning them that face masks must be worn and distance between them and others must be kept. When Hungary’s total number of vaccinated people reached 3 million in early April, the government lifted restrictions gradually. Finally, in June 2021, the third wave ended (see the timeline in Fig. 1).

### Database of urban parks and park classification

We used Urban Atlas 2018 (UA 2018), provided by Copernicus Land Monitoring Service, to identify green urban areas and parks in Hungarian settlements. The database contains information on Hungary’s 19 functional urban areas (FUAs), which are as follows: Békéscsaba, Budapest, Debrecen, Dunaújváros, Eger, Győr, Kaposvár, Kecskemét, Miskolc, Nyíregyháza, Pécs, Sopron, Szeged, Székesfehérvár, Szolnok, Szombathely, Tatabánya, Veszprém, and Zalaegerszeg. Then, we selected the “green urban areas” category marked with the code “14100” as the target area of our analysis. The selection process in ESRI ArcGIS 10.5 software resulted in 4612 polygons. Adjacent polygons were merged to form a continuous green area if the distance between two or more polygons were smaller than 10 m.

We exported the resulting 1888 polygons in shapefile format and performed a spatial data query from the mobile device location database; we then created a filtered dataset in two phases. First, we took all the location data points from the raw dataset within the determined polygons. Second, we took all the devices that produced these location data points and created a simple table of their general activities. Ultimately, we compiled a dataset of more than 28 million mobile device location data points for 1884 parks in 191 settlements over the two years the research covers (see the mobile device location database’s description in the next section). We then classified the parks into five categories based on their sizes. We reviewed some international examples to define park categories (including Park Classification by Dallas Park & Recreation: https://www.dallasparks.org/151/Park-Classifications), which helped us create a tailored park classification (Table 1).

**Table 1:** Summary statistics of urban parks by park category.

Park class	Park size range (hectare)	Number of parks	Total area (hectare)	Average area per park (hectare)	Total number of devices*	Total number of location data points	Share of parks in Budapest (%)	Share of parks in suburban settlements in the BA (%)	Share of parks in large cities (%)
Metropolitan park	Over 50.01	18	1814.11	100.78	402,868	5,977,251	55.56	5.56	38.89
Community park	10.01–50.00	146	2779.03	19.03	446,609	5,416,260	34.25	23.29	42.47
Neighborhood park	3.01–10.00	401	2114.03	5.27	764,941	7,050,010	23.94	34.16	41.90
Block park	1.01–3.00	781	1406.57	1.80	816,075	6,664,135	21.90	36.24	41.87
Minipark	0.20–1.00	538	340.01	0.63	426,937	3,089,887	23.79	23.61	52.60

*The dataset consisted of a total of 666,156 distinct mobile devices, indicating that some devices appeared in more one than parks on the same day.

**Figure 2:**
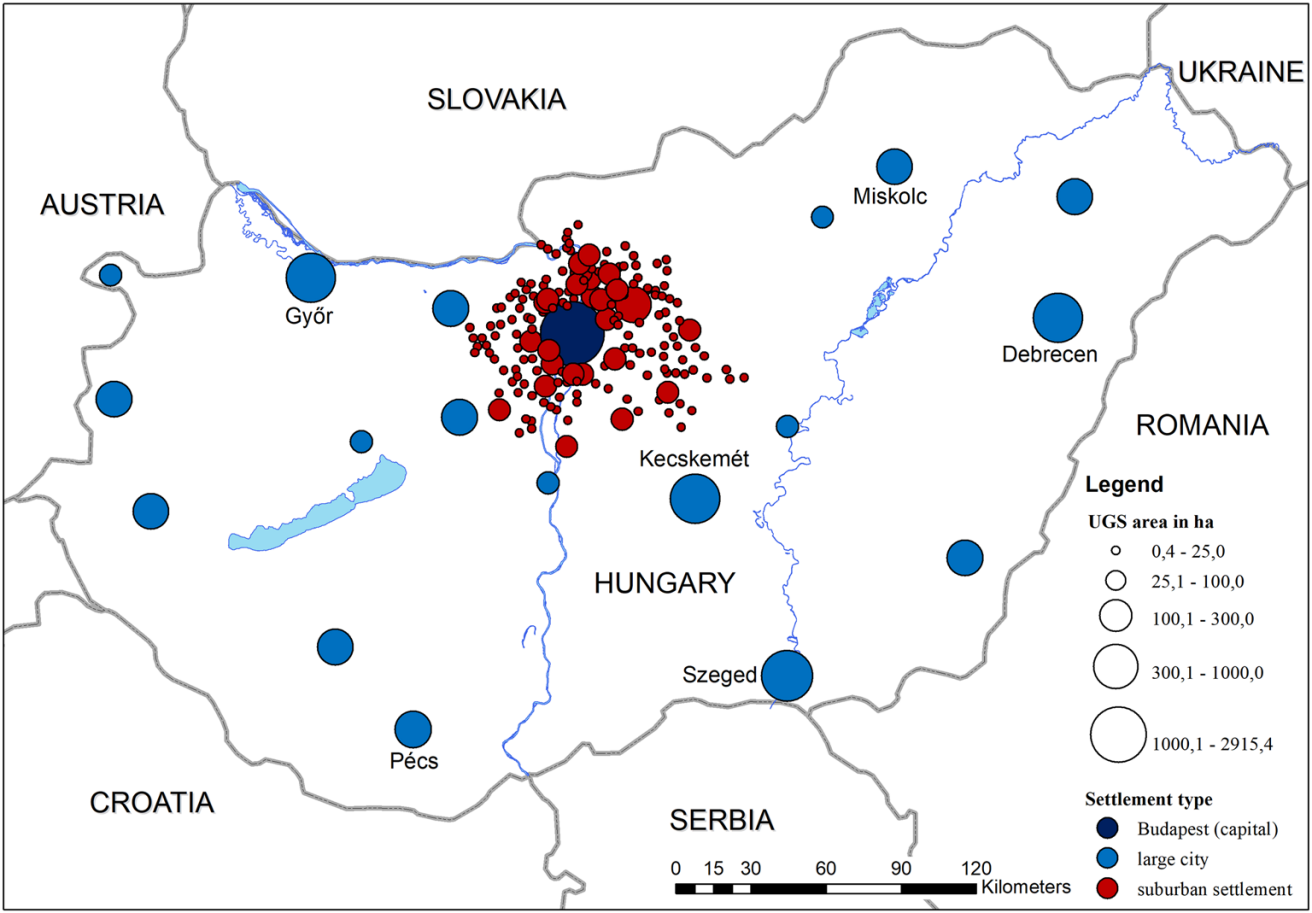
Settlement types and aggregated park areas by settlement. For Hungary, UA 2018 contains information on urban green spaces located in 19 FUAs. The Budapest FUA can be divided into two parts: the capital city of Budapest and a zone of suburban settlements. Large cities in the countryside have underdeveloped agglomerations. While these 191 settlements account for 6.05% of Hungary’s settlements, they are home to 47.54% of the population. The map was generated using ArcGIS Desktop 10.8 (https://www.esri.com/en-us/arcgis/products/arcgis-desktop/overview).

To understand spatial patterns in park visitation, we aggregated the 191 settlements across Hungary into three settlement types, depending on each one’s location and hierarchical position in the urban network. The data content of UA 2018 significantly influenced the creation of settlement types. Hungary has 3155 settlements (cities, towns, and villages), but UA 2018 only contains land use and land cover data for FUAs. An FUA consists of a municipality with at least 50,000 inhabitants and its commuting area. The largest FUA in terms of population and the number of settlements is the BA which we divided into the central city and a zone of suburban settlements surrounding it. Therefore, the settlement types in the research are as follows (Fig. 2): (1) Budapest (the central city of the BA), the BA (which consists of 169 suburban settlements where we found park visit data), and large cities in the countryside. Finally, we aggregated data on urban parks by settlement type (Table 2).

**Table 2:** Summary statistics of urban parks by settlement type.

Settlement type	Number of settlements	Population	Average population per settlement	Number of parks	Total area of parks (hectare)	Average area of parks (hectare)	Number of devices*	Number of location data points
Budapest (capital)	1	1,662,438	1,662,438	455	2915.44	6.41	267,261	2,679,705
Suburban settlements in the Budapest Agglomeration	168	1,229,599	7319	582	2002.48	3.44	1,769,996	16,474,476
Large cities	19	1,720,497	90,552	847	3535.83	4.17	820,173	9,043,362

*The dataset consisted of a total of 666,156 distinct mobile devices, indicating that some devices appeared in more one than parks on the same day.

### Mobile device GNSS-location database

The processed data, including the filtering, are stored in a trusted repository^27^. Because the data come from a proprietary and sensitive dataset acquired from UberMedia Inc. (a data intelligence company now part of Near Intelligence Holdings Inc.), we uploaded and archived them in an encrypted form. The raw form contains 5.2 million unique devices and 3.18 billion location data points covering Hungary between June 2019 and June 2021. Parsing, processing, and publishing have been done using open-source software^28^, and all code used is located in the cited archives with linked repositories. As a result of the spatial query, we obtained a dataset of 28,197,543 mobile device location data points produced by 666,156 individual devices. In 2020, approximately 6.93 million people used smartphones in Hungary^29^. Therefore, although one person can have and use more than mobile devices, our park-visiting data represents 10% of the smartphone user population, the same rate described in a US study^30^.

## Results

### Park visitation trends by park category

#### Comparison of park visitation trends in the inter-wave period of 2020 over the same period of 2019 by park category

As Table 3 shows, in the inter-wave period of 2020, most park categories witnessed a roughly 10–15% increase in the number of devices (i.e., users) compared to the pre-Covid-19 period of 2019. However, with a significant decline in the number of devices (− 35.53%), metropolitan parks (i.e., those larger than 50.01 hectares) are considered exceptions. Reviewing the change in location data points, we can realize that community parks (10.01–50.00 hectares) have the highest increase. Based on the difference in the number of location data points by park category, we can conclude that the smaller the park is, the lower the increase in the number of location data points was. For example, location data points increased by almost 61% for community parks but only 23% for miniparks. When comparing the number of location data points collected in the pre-Covid-19 period with those in the inter-wave period of 2020, only metropolitan parks experienced a decline (− 14.64%). The number of location data points/devices increased in all cases, but for miniparks to the least extent.

**Table 3:** Comparison of park visitation trends in the inter-wave period of 2020 with the pre-Covid-19 period of 2019 by park category, based on mobile device location data.

	Number of parks	Devices/day/parks			Location data points/day/parks			Location data points/devices		
		Pre-Covid-19 period of 2019	Inter-wave period of 2020	Change (%)	Pre-Covid-19 period of 2019	Inter-wave period of 2020	Change (%)	Pre-Covid-19 period of 2019	Inter-wave period of 2020	Change (%)
Metropolitan parks	18	40.93	26.39	− 35.53	553.32	472.30	− 14.64	13.52	17.90	+ 32.39
Community parks	146	4.43	4.99	+ 12.72	41.95	67.51	+ 60.93	9.48	13.53	+ 42.77
Neighborhood parks	401	2.69	2.98	+ 10.95	20.42	30.05	+ 47.12	7.60	10.08	+ 32.60
Block parks	781	1.47	1.69	+ 14.95	10.24	14.53	+ 41.88	6.98	8.61	+ 23.42
Miniparks	538	1.12	1.26	+ 12.28	7.59	9.33	+ 23.06	6.75	7.40	+ 9.60

#### Comparison of park visitation trends in Waves 2–3 of the Covid-19 pandemic (2021) over Wave 1 (2020) by park category

Unsurprisingly, during the first wave of the pandemic in 2020, parks experienced a significant decline in visitation compared to the pre-Covid-19 period. However, comparing park visitation trends in the first wave with the second to third may give us a new insight into how people used the parks in different phases of the Covid-19 pandemic.

Table 4 shows that in Waves 2–3, each park class (except for metropolitan parks) experienced a 12–16% decline in the number of devices (i.e., users) compared to Wave 1. For metropolitan parks, the number of devices decreased by almost 59%. During Wave 1, 55,762 devices appeared in the 18 metropolitan parks and 49,630 in the 146 community parks; in Waves 2–3, community parks hosted a total of 43,749 devices compared to the 22,932 devices in metropolitan parks.

**Table 4:** Comparison of park visitation trends in Waves 2–3 of 2021 with Wave 1 of 2020 by park category, based on mobile device location data.

	Number of parks	Devices/day/parks			Location data points/day/parks			Location data points/devices		
		Wave 1 in 2020	Waves 2–3 in 2021	Change (%)	Wave 1 in 2020	Waves 2–3 in 2021	Change (%)	Wave 1 in 2020	Waves 2–3 in 2021	Change (%)
Metropolitan parks	18	37.78	15.54	− 58.88	470.81	209.76	− 55.45	12.46	13.50	+ 8.34
Community parks	146	4.15	3.65	− 11.85	47.67	41.10	− 13.78	11.50	11.25	− 2.19
Neighborhood parks	401	2.57	2.17	− 15.83	22.04	16.51	− 25.08	8.56	7.62	− 10.98
Block parks	781	1.35	1.14	− 15.99	10.40	8.43	− 18.92	7.69	7.42	− 3.48
Miniparks	538	0.98	0.85	− 14.15	7.52	4.79	− 36.26	7.64	5.67	− 25.76

The amount of location data points also declined for each park category in Waves 2–3 compared to Wave 1. Waves 2–3 produced significantly fewer location data points in metropolitan parks and miniparks than in Wave 1 (− 55.45 and − 36.26%, respectively). Compared to Wave 1, the number of location data points/devices increased by 8.34% for metropolitan parks and decreased by 25.76% for miniparks in Waves 2–3. Finally, the number of location data points/devices also declined for other park classes.

### Park visitation trends by settlement type

#### Comparison of park visitation trends in the inter-wave period of 2020 over the same period of 2019 by settlement type

After investigating park visitation trends by park category, we demonstrate the changes in park visitation through the lens of settlement types. As Table 5 shows, park visitation in terms of the number of devices increased for each settlement type during the inter-wave period of 2020, compared to the pre-Covid-19 period. While Budapest and large cities across the country experienced less than a 2% increase in the number of devices that appeared in parks, settlements in the Budapest Agglomeration (BA) faced explosive growth in park visitation. The magnitude of the change seems more dramatic if we consider the increase in the location data points/days/parks, which reached 133% for settlements in BA.

**Table 5:** Comparison of park visitation trends in the inter-wave period of 2020 with the pre-Covid-19 period of 2019 by settlement type, based on mobile device location data.

	Number of parks	Devices/day/parks			Location data points/day/parks			Location data points/devices		
		Pre-Covid-19 period of 2019	Inter-wave period of 2020	Change (%)	Pre-Covid-19 period of 2019	Inter-wave period of 2020	Change (%)	Pre-Covid-19 period of 2019	Inter-wave period of 2020	Change (%)
Budapest	582	5.58	5.65	+ 1.40	48.85	55.73	+ 14.07	8.76	9.86	+ 12.49
Budapest agglomeration*	455	0.59	0.81	+ 38.36	3.94	9.20	+ 133.39	6.71	11.32	+ 68.68
Large cities**	847	1.57	1.59	+ 1.31	13.97	18.97	+ 35.81	8.88	11.90	+ 34.05

*Not including Budapest.

**Cities with county right.

#### Comparison of park visitation trends in Waves 2–3 of the Covid-19 pandemic (2021) over Wave 1 (2020) by settlement type

In the inter-wave period of 2020, parks experienced an increase in visitation over the same period of 2019, irrespective of whether they were located in Budapest, suburban settlements, or large cities. In contrast, in Waves 2–3, park visitation in terms of the number of devices and location data points significantly declined, compared to Wave 1.

In Waves 2–3, the number of location data points/devices in parks decreased for each settlement type, but the highest decrease was registered (i.e., with 17.77%) for suburban settlements in the BA (Table 6).

**Table 6:** Comparing park visitation trends in Waves 2–3 of 2021 with Wave 1 of 2020 by settlement type, based on mobile device location data.

	Number of parks	Devices/day/parks			Location data points/day/parks			Location data points/devices		
		Wave 1 in 2020	Waves 2–3 in 2021	Change (%)	Wave 1 in 2020	Waves 2–3 in 2021	Change (%)	Wave 1 in 2020	Waves 2–3 in 2021	Change (%)
Budapest	582	5.41	4.19	− 22.57	45.48	32.82	− 27.83	8.40	7.83	− 6.79
Budapest agglomeration*	455	0.65	0.60	− 8.45	6.57	4.95	− 24.72	10.10	8.31	− 17.77
Large cities**	847	1.25	0.91	− 27.45	14.07	9.15	− 35.01	11.23	10.06	− 10.42

*Not including Budapest.

**Cities with county right.

## Discussion

Urban green spaces are often regarded as nature-based solutions offering innovative approaches to increase the quality of urban settings, mitigate the urban heat island effect, enhance local resilience, promote sustainable lifestyles, and improve both the health and the social well-being of residents^31–34^. Furthermore, urban green spaces provide cultural ecosystem services for people allowing them to relax in nature even in densely built urban areas.

During the Covid-19 pandemic, when governments significantly restricted people’s movement, the value of locally available urban green spaces dramatically increased. For example, a line of studies has demonstrated the positive impacts of urban green spaces on visitors’ physical and mental health and well-being compared to those who did not visit such amenities^35–37^. However, it is well-documented that park visitation trends changed significantly in subsequent phases of the Covid-19 pandemic. In the early stage of the pandemic in 2020, when governments across the world imposed total lockdowns and curfews, visitation to parks declined compared to the pre-pandemic period^7,38–40^. After lifting restrictions and launching nationwide mass vaccination programs, park visitation has gradually recovered to the pre-pandemic level^20^. To study changes and fluctuations in park visitation trends, researchers worldwide tended to apply new methods, including analyzing mobile device location data^22,23,37,41–43^.

Overall, in this paper, we wanted to study park visitation trends in four different phases of the Covid-19 pandemic in Hungary. First, we compared park visitation trends in the inter-wave period of 2020 over the same period of 2019 by park category. We found that metropolitan parks (i.e., those larger than 50.01 hectares) experienced significant decline in visitation, while smaller parks, particularly community parks, were visited by more people. To explain this pattern, we must review some national tourism data. In 2019, Hungarian people were involved in 24.86 million international trips, of which 7.62 million took place in the third quarter (i.e., July–September). Domestic trips reached 4.85 million in the same period, making only 64% of the international ones. In the spring of 2020, many European countries closed their borders to foreign citizens to prevent the coronavirus’s spread. This measure was a significant setback for international tourism. When the daily deaths caused by Covid-19 decreased significantly, countries gradually reopened their borders. However, as the UN World Tourism Organization (UNWTO) reported, international tourist arrivals worldwide fell by 72% in January–October of 2020 from the previous year, representing 900 million fewer international tourist arrivals^44^.

**Figure 3:**
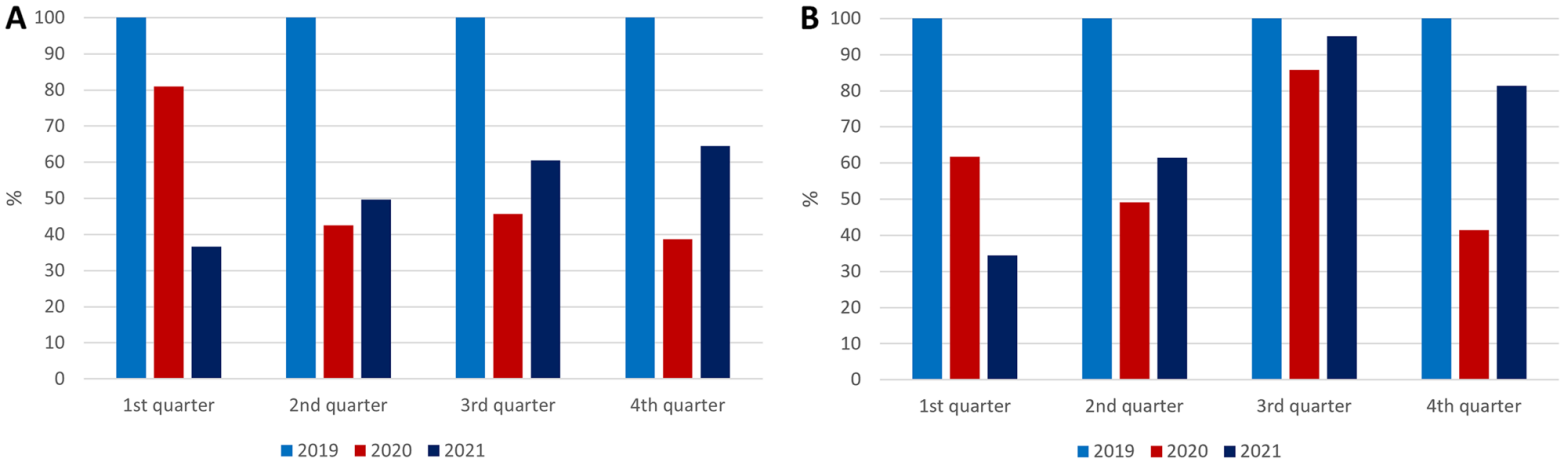
Change in the number of international (**A**) and domestic trips (**B**) in 2020 and 2021, compared to the base year of 2019 (100%). Although most European countries opened their borders to tourists in the summer of 2020, people feared getting infected with the coronavirus during the vacation, resulting in a significant setback for international tourism. In the third quarter of 2019, Hungarian people participated in 7.62 million international trips, which fell to 3.48 million in that quarter of 2020. In contrast, domestic tourism experienced a moderate decline: from 4.85 million domestic trips taken in the third quarter of 2019, it fell to 4.16 million in the same quarter of 2020. Data show that those who participated in tourism in the summer of 2020 visited domestic tourism destinations rather than abroad. However, most people preferred to stay at home during the inter-wave period.

Although most restrictions regarding international tourism were lifted in 2020, Hungarian government officials (including the Prime Minister) and health professionals strongly advised people not to leave Hungary in the summer and recommended preferring domestic tourism. As a result, compared to the summer of 2019 (third quarter), the number of international trips in 2020 decreased by 54.33%, while the number of domestic trips showed a decline of 14.29% (Fig. 3). That is, most people either spent their vacation in Hungary or did not participate in tourism at all.

We assume that the number of devices and location data points increased in most parks because people who did not leave their town during the summer of 2020 spent more time in the parks. This change is also reflected in location data points/devices, which increased for all park categories (i.e., people used their smartphones for a longer time in the parks). The highest increase in the number of location data points was experienced for community parks, suggesting that people preferred to visit spacious and well-equipped parks rather than smaller ones. One may ask why there was a decline in the average number of devices and location data points in metropolitan parks in the inter-wave period of 2020 over the same period of 2019 if they had the largest area and provided the most ecosystem services. For example, in 2019, 13 times more location data points were produced per day in metropolitan parks than in community parks (553.32 vs. 41.95), while this difference was only seven times larger in 2020 (472.30 vs. 67.51). We found two reasons that may explain this trend.

**Figure 4:**
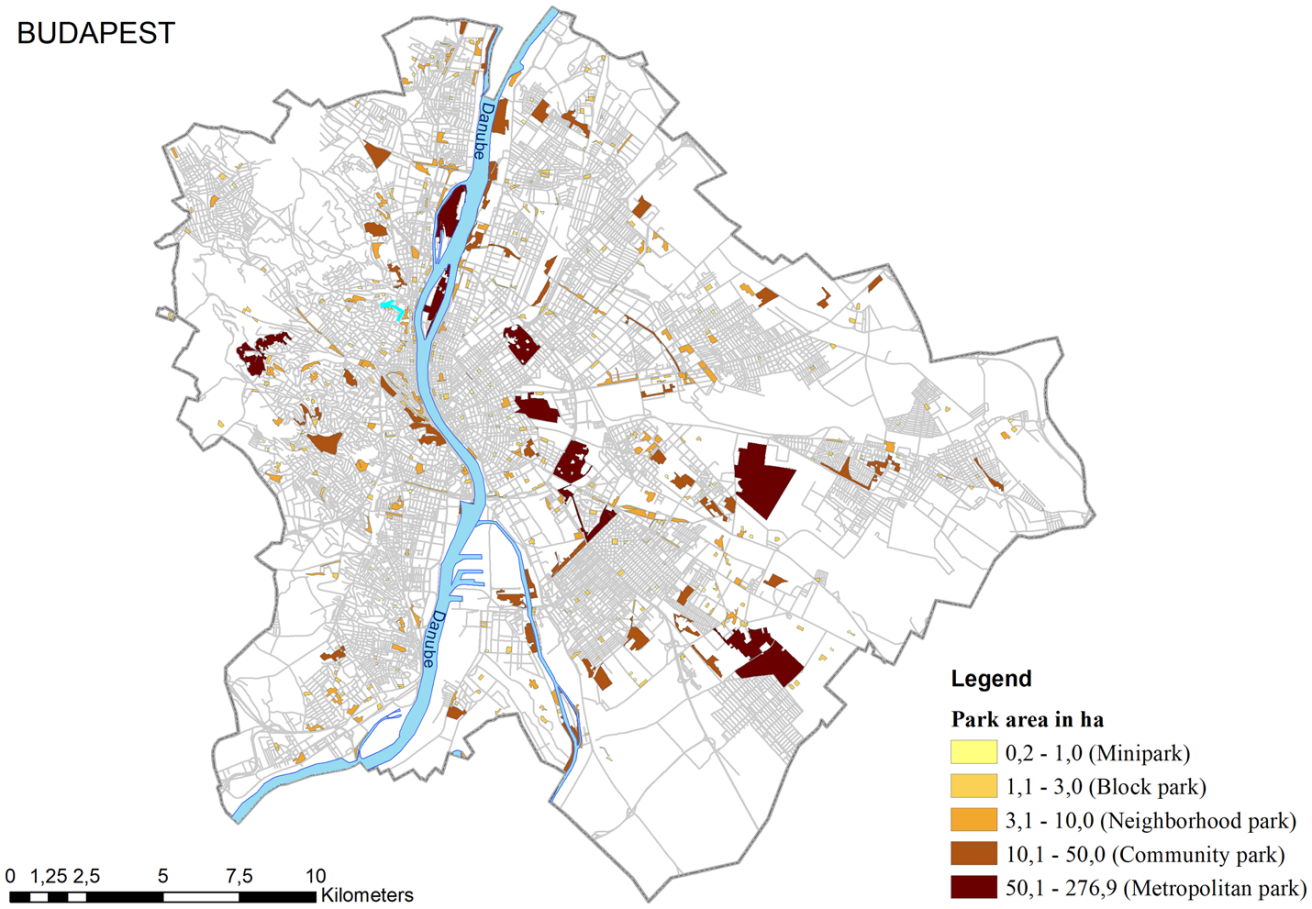
Location of urban parks and other urban green spaces in Budapest. Metropolitan parks are either relatively peripheral or isolated geographical positions in the city. Usually, the easiest way to access these parks is by public transportation. In contrast, community parks are more integrated into the urban fabric, making them easier to access on foot. In addition, most community parks are well-equipped with amenities, and they are large enough to allow people to keep their distance from others. The map was generated using ArcGIS Desktop 10.8 (https://www.esri.com/en-us/arcgis/products/arcgis-desktop/overview).

First, metropolitan parks, with an average size of approximately 100 hectares, are often located at the edges of cities or relatively far from residential areas with high populations (see Fig. 4). Accordingly, the easiest way to access a metropolitan park is usually by public transportation. Yet in the summer of 2020, right after the pandemic’s first wave, people avoided being enclosed with dozens of other people in small spaces like buses or trams. We thus assume that fear strongly influenced people’s decision not to visit metropolitan parks. In contrast, community parks with an average size of 19 hectares are located in or close to residential areas and easily accessed on foot. Moreover, a community park remains large enough to ensure people can maintain social distancing. Second, metropolitan parks generally accommodate many summer events like festivals, fairs, and outdoor sporting events, most of which were canceled in 2020, resulting in a formidable setback for park visitation. For example, the Sziget Festival—one of Europe’s largest music and cultural festivals organized annually on the Óbuda-sziget, a 108-hectare island on the Danube—was visited by 530,000 people in 2019. However, the Covid-19 pandemic led to the event’s cancellation in 2020 (also in 2021)^45^.

We also compared park visitation trends in Waves 2–3 of the Covid-19 pandemic (2021) over Wave 1 (2020) by park category. We concluded that during Waves 2–3, parks were visited by fewer people and for a shorter time than in Wave 1, except for metropolitan parks, which were also used by fewer people who spent a slightly longer time in the parks.

A contradiction may seem to occur here: during Wave 1, the national government imposed a strict nationwide lockdown and curfew, and local governments across the country closed most urban parks, preventing people from meeting each other in a public open space. Meanwhile, the media advised people to stay at home. In contrast, during Waves 2–3, the government tried to avoid fully locking down the country and imposed only a night curfew. Additionally, health professionals advised people to visit parks because it would benefit their physical and mental health during such a stressful period. However, although people were allowed and encouraged to visit the parks, park visitation significantly declined.

We found a significant difference in the characteristics of Wave 1 and Waves 2–3 that might explain the differences between the park visitation trends in the Covid-19 waves of 2020 and 2021. During Wave 1, 482 deaths associated with Covid-19 were registered, and the daily deaths peaked at 17 people (April 20, 2020), lower than the average daily deaths related to influenza in a regular winter^46^. However, in Waves 2–3 (especially in Wave 3), the number of daily deaths explosively increased (see Fig. 1), reaching a peak of 311 deaths on April 7, 2021 (for example, on April 7 and 8, 21% more Covid-19 associated deaths were registered than in Wave 1 in total). The daily death statistics ran in the headlines for months, and endless debates weighed whether the government introduced adequate measures to stop the spread of Covid-19. Consequently, many regarded the parks as unsafe places where they could easily be infected with the coronavirus (see also Bristowe & Heckert^3^).

Metropolitan parks experienced the highest decline in the number of devices and location data points, suggesting that people avoided visiting the largest parks in the city. In the previous section, we highlighted that metropolitan parks are generally located in a geographically peripheral or relatively isolated position^47^, hindering most people from accessing them on foot or by bike. However, compared to Wave 1, the number of location data points/devices increased by 8.34% in Waves 2–3, meaning that people who visited metropolitan parks spent slightly more time there. A likely cause is that large metropolitan parks (e.g., the People’s Park and the City Park, with an area of 129 hectares and 120 hectares, respectively) are equipped with several walking paths, bike paths, running tracks, and Nordic walking tracks. Thus, we may conclude that the occasional park users disappeared from metropolitan parks, whereas the regular park users spent slightly more time there. In contrast, the number of location data points/devices significantly decreased for miniparks, suggesting that in Waves 2–3, people spent much less time there than in Wave 1.

Following the demonstration of how park visitation trends changed in different pre-pandemic and Covid-19 phases by park category, we investigated park visitation trends by settlement type (i.e., Budapest, suburban settlements around Budapest, and large cities in the country). We found that park visitation increased for each settlement type during the inter-wave period of 2020, compared to the pre-Covid-19 period. However, while Budapest and large cities across the country experienced a moderate increase in the number of devices that appeared in parks, settlements in the Budapest Agglomeration (BA) faced explosive growth in park visitation.

To explain these trends, we must understand the functional relationship between Budapest and settlements in the BA. Home to nearly 2.63 million people, the BA is one of the largest urban areas in the European Union^48^. Budapest has a population of 1.75 million, and 80 cities, towns, and villages in its suburban zone have a total population of 880,000. Naturally, most people living in the suburban zone of the BA (approximately 500,000 people) commute to Budapest every day to work or study.

However, the Covid-19 pandemic engendered radical changes in the socioeconomic and spatial mechanisms of the BA. In 2019, before the arrival of Covid-19, only 5.4% of full-time employees in Budapest worked primarily from home; this share was less than 2% for suburban settlements on average. When the pandemic hit the country, many Budapest-based companies operating in, for example, finance, media, information and communication technology, transferred themselves to remote work. Therefore, most employees living in the suburban zone were no longer required to commute to Budapest daily. In 2020, 21.3% of full-time employees in Budapest worked remotely, many of whom lived in suburban settlements of the BA. Furthermore, when rumors about the possible infiltration of Covid-19 and the subsequent lockdowns started to circulate among people at the beginning of 2020, many residents of Budapest desperately sought to buy a house in suburban settlements (and across the country) to move there temporarily or permanently^49^. Some small villages around Lake Balaton, characterized by steady population loss for decades, witnessed a 20% population growth in 2020, whereas real estate prices in other villages in the countryside skyrocketed by 400%. Some newspapers characterized this process as an exodus from Budapest.

Finally, we must add that people tended to stay home in the summer of 2020 rather than spend the vacation elsewhere. Thus, the surge in the daily population of the suburban settlement also reflects the increase in park visitation.

In the inter-wave period of 2020, park visitation in terms of the number of devices in Budapest and large cities hardly changed compared to the previous year because many people spent much of their summer vacation in the countryside or feared using the parks. However, based on the increase in location data points/devices, we can conclude that those who visited the parks spent more time there than before the pandemic.

If we compare park visitation trends in Waves 2–3 of the Covid-19 pandemic (2021) over Wave 1 (2020) by settlement type, we can realize that suburban settlements experienced the highest decrease in the number of location data points/devices (i.e., people tended to spend a much shorter time in the parks than before). Surprisingly, the decline of park visitation in terms of the number of devices and location data points in suburban settlements was not as high as for Budapest and large cities. To explain the above pattern, we must review the average sizes of parks by settlement type. For example, in Budapest, the average size of parks is 6.4 hectares, while in large cities and suburban settlements, it is 4.2 and 3.4 hectares, respectively. Considering the distribution of parks in different sizes by settlement type, we realize that suburban settlements host hardly any metropolitan parks with more than 50.01 hectares, whereas 55.56 and 38.89% of such parks are located in Budapest and large cities, respectively.

Moreover, only 23.29% of the community parks (i.e., those having a size of 10.01–50.00 hectares) can be found in suburban settlements. Thus, suburban settlements are typically home to medium-sized and small parks that generally host only a few ecosystem services (e.g., a playground, a dog walking path, and an outdoor gym). For example, in Waves 2–3, miniparks in suburban settlements experienced a decline of 42.44% in the number of location data points/devices compared to Wave 1. At the same time, in Budapest, where even these small urban green spaces are well-equipped, the decline was only 6.06%.

The findings of this research shed light on the constraints of the usability of large and miniparks in pandemic situations and demonstrate the increased importance of medium-sized urban parks. Metropolitan parks (i.e., very large urban parks) are excellent locations for urban events, sports, and recreation^50,51^, while mini- or pocket parks provide access to green spaces in dense inner cities^52–55^. However, our findings suggest that metropolitan parks (because of their peripheral location and relative isolation) and miniparks (because their small size hinders people from keeping their distance from others) fail to work adequately during a pandemic. While acknowledging the benefits of mini- and pocket parks in dense urban areas (see, for example, Liu and Wang^56^), we recommend that cities focus instead on creating a network of medium-sized parks with extensive integration into the urban fabric. Nevertheless, the accessibility of existing large parks should also be increased.

Another important lesson from this study is that large cities may act as population emitters during crises, suddenly burdening suburban settlements and rural towns’ supply systems, especially those with waterfronts^57^. Unfortunately, in Hungary, the government was unprepared for this eventuality. Thus, measures to halt the outmigration from large cities were implemented when problems had already escalated. Further compounding these issues, although hotels were required to close, rural tourism accommodation providers were allowed to still receive guests in large numbers, often leading to conflicts with the local population^57^. Therefore, the rules affecting the movement of people in such situations as the Covid-19 pandemic must be made in time and precisely so they can reach their goals.

### Limitations

Finally, some limitations of the study must be mentioned. Our sample consists of urban centers and suburban settlements, so we have no information about changes in park visitation in small towns and villages in rural areas. Nevertheless, because natural areas (e.g., forests) are more easily accessible from small settlements in the countryside, the shutdown of urban parks probably did not hinder local people from accessing nature.

We also do not have data on mobile device users’ social characteristics and demography. We only have indirect data on these through an analysis that compared location data used for this study and aggregated telecommunication data bought from a local service provider (LSP). We came to the following conclusions: the GNSS location data may underrepresent those who have a gender that is not known by the LSP or are categorized as low income by the LSP. On the other hand, those on a roaming plan might be somewhat overrepresented, and so can those in their home at the time of the location data recorded. However, the representations seem stable across age groups, and these observations do not seem to change over time. Our analysis did not show the scale of these biases to be especially worrying, but this method is very limited due to the difference of magnitude between the samples in the data sources. According to another Hungarian research^58^, active smartphone use is typical for younger people (or less usual for older people), which is a conflicting finding with our analysis. Thus, older people are probably underrepresented in the applied database (probably similar to all mobile GNSS databases internationally). Thus, a dedicated study would be valuable to explore location data’s representativity.

A further limitation is that in the database, both the devices and the number of mobile GNSS location data fluctuate widely daily; therefore, we used an analysis technique based on more extended periods to smooth them out and paid attention to seasonality.

Another critical point can be the location data positional accuracy, about which we do not have any specific information from Near/UberMedia Inc. However, the longitude and latitude data in the database were provided with six decimal places (1/1,000,000 degree), meaning that they can define a location with sub-meter accuracy. Conversely, the accuracy of the location data collected with mobile phones falls significantly short of this. According to a study by Zandbergen and Barbeau^59^, the median horizontal error of A-GPS measurements was between 5 and 8.5 m and never exceeded 30 m. According to a study conducted in an urban, high-multipath environment^60^, the average horizontal position accuracy varied between 7 and 13 m. On the one hand, this accuracy is suitable for our analysis since the dedicated minimum mapping unit of the Urban Atlas database is 0.25 hectares (2500 square meters). On the other hand, we could not use stay (stop) detection because a stop does not correspond to a point but a radius, meaning that we could lose valuable spatial accuracy, which can be critical in this use case since many different points of interest (e.g., public transport stops and restaurants) are located right next to parks.

Finally, it is essential to note that we did not have a dataset covering a single year before the Covid-19 pandemic, so we could not fully assess the baseline of park visitation trends in Hungarian parks.

## Conclusions

In this study, we used mobile device location data to explore park visitation trends in Hungary during the Covid-19 pandemic. The findings indicate that park visitation exhibited distinct changes throughout the pandemic. Specifically, park visitation increased during the inter-wave period of 2020 compared to the pre-pandemic period of 2019. However, there was a subsequent decline in park visitation during Waves 2–3 of 2021, compared to the first wave of 2020. These findings highlight the dynamic nature of park visitation trends, influenced by the evolving circumstances and phases of the pandemic.

Our study demonstrated that park visitation during the Covid-19 pandemic varied across park and settlement types. While the smallest and the largest parks experienced a decline in visitors, mid-sized parks attracted more people. The finding suggests that people in pandemic times tend to visit urban parks that are highly integrated into the urban fabric but large enough to allow visitors to keep the distance from others. Furthermore, park visitation significantly increased in suburban settlements, contrary to Budapest and large cities where overall park visitation decreased. Disparities in visitation trends by settlement type emphasize the importance of considering local context and demographics when planning and managing urban parks.

In follow-up research, we will examine the catchment areas of urban parks before, during, and after the Covid-19 pandemic using mobile device location data. By determining the so-called home positions of visitors, our primary goal is to find out whether the pandemic has changed the socioeconomic and demographic composition of visitors and whether it has expanded or narrowed the area of attraction of the parks. In addition, we will conduct a multiple regression analysis to explore whether different factors (e.g., location, size, and accessibility) are statistically significant.

## Supplementary Information

Supplementary Table 1.

## Data Availability

Urban Atlas 2018 (UA 2018) is freely available after registration on Copernicus Land Monitoring Service’s website: https://land.copernicus.eu/. The raw data supporting the findings of this study are commercially available from Near Intelligence Holdings Inc. Due to this, restrictions apply to the availability of the data, which were used under the general terms and conditions defined by UberMedia Inc. (now part of Near Intelligence Holdings Inc.). The raw data used in this study cannot be shared with third parties. However, the derivative data are available from the authors upon reasonable request. The software used for processing raw data is available at the following link: https://doi.org/10.5281/zenodo.7644587.
