# Supplementary material for: Exploring park visitation trends during the Covid-19 pandemic in Hungary by using mobile device location data
Source: Sci Rep. 2023 Jul 8;13:11078. doi: 10.1038/s41598-023-38287-3 (PMC10329667; doi:10.1038/s41598-023-38287-3)
Supplement: Supplementary file 1 — Supplementary Table 1. [file 41598_2023_38287_MOESM1_ESM.pdf]

Supplementary Table S1

Chronological summary of events and governmental measures during the COVID-19 pandemic in Hungary between March 4, 2020, and June 1, 2021

Period	Events and measures	Messages from government officials, health professionals and the media.	Deaths from Covid-19
First wave<br><br>March 4, 2020 – May 24, 2020	March 4, 2020: First Covid-19 case detected in Hungary.<br>March 11, 2020: Government's announcement about the imposition of state of emergency.<br>March 16, 2020: Closure of schools and universities and introduction of remote teaching.<br>March 17, 2020: Closure of borders to foreign citizens, ban on events and public gatherings, and closure of urban green spaces.<br>March 27 – April 11, 2020: Imposition of countrywide curfew.<br>April 27, 2020: Compulsory face mask wearing.<br>May 4, 2020: Curfew is lifted in the countryside, restaurants are allowed to open outdoor terraces, weddings and funerals are allowed to be held with limited participants.<br>May 18, 2020: Curfew is lifted in Budapest, restaurants are allowed to open indoor rooms, hotels may reopen.<br>May 24, 2020: The first wave ends.	Stay at home!<br><br>Do not leave your home if not necessary!<br><br>Wear a face mask!	Total deaths: 482 people<br><br>Average daily deaths:<br>5.88 people<br><br>Daily death peak:<br>17 people (April 20)
Interim period<br><br>May 25, 2020 – November 10, 2020	May 25, 2020: Kindergartens and childcare centers may reopen in the countryside.<br>June 1, 2020: Kindergartens and childcare centers may reopen in Budapest.<br>June 2020: Travel restrictions are gradually lifted.<br>July 2020: Evolving second wave of Covid-19 worldwide. Classification of countries into different risk categories (the low-risk category contains mostly European countries).<br>September 2020: Start of the second wave. No strict restrictions are imposed.<br>September 1, 2020: Closure of the borders. 14-day quarantine for those enter in the country.<br>September 2020: Schools remain open, but classes where a Covid-19 case is detected must be quarantined for 14 days.	Travelling abroad for vacation is allowed, but it is highly advised to rather spend the summer in Hungary.	Total deaths: 2,114 people (40 percent in November)<br><br>Average daily deaths:<br>12.44 people<br><br>Daily death peak:<br>107 people (November 7)
Second and third waves<br><br>November 11, 2020 – May 31, 2021	November 11, 2020: Curfew from 8 p.m. Public gatherings are not permitted, restaurants must be closed, shops and supermarkets are allowed to be open until 7 p.m., high schools (from 9th grade) and universities must change to remote teaching, museums, swimming pools, libraries, zoos, and ice rinks must be closed, events must be cancelled.<br>December 26, 2020: Vaccination of health care workers starts.<br>February 2021: Mass vaccination with all kinds of vaccines starts.<br>February 2021: Prior to the ebb of the second wave, a third wave starts.<br>March 2021: Surge in daily deaths.<br>March 8, 2021: Closure of kindergartens and elementary schools.<br>March 2021: Government announces that if the number of vaccinated people reaches 2.5 million, lockdowns and restrictions may gradually be lifted.<br>April 7, 2021: Daily death peak of 311 people.<br>April 7, 2021: Time of night curfew shortens. Shops and supermarkets are allowed to reopen.	Avoid crowded areas!<br><br>Keep distance between yourself and others!<br><br>Wear a face mask!	Total deaths: 27,137 people<br><br>Average daily deaths:<br>134.34 people<br><br>Daily death peak:<br>311 people (April 7)

	April 19, 2021: Reopening of childcare centers, kindergartens, and elementary schools.<br>April 24, 2021: Having been vaccinated a population of 3.5 million restaurants may reopen outdoor terraces.<br>May 1, 2021: Having been vaccinated a population of 4 million the nightly curfew is shortened, shops and supermarkets may be open for a longer time, and museums, swimming pools, libraries, zoos, and ice rinks are allowed to be visited by those having vaccination certificate.<br>May 23, 2021: Having been vaccinated a population of 5 million the nationwide curfew is lifted, people are not required to wear a face mask on public open spaces, and events with not more than 500 people are allowed to be held on open spaces.<br>June 1, 2021: The third wave ends.		
